# Improving quality indicators for the treatment of acute myocardial infarction: impact of the disease-specific care certification

**DOI:** 10.1186/cc12627

**Published:** 2013-06-19

**Authors:** PG Melo de Barros e Silva, MY Okada, S Simoes, VA Fernandes, TA Macedo, DL Ramos, MJ Rodrigues, MC do Amaral Baruzzi, V Furlan

**Affiliations:** 1 Hospital Totalcor, Cerqueira César, São Paulo, SP, Brazil

## Introduction

Registries have shown that there still exists a large gap between what is recommended by evidence-based guidelines and what is actually offered to the patient in clinical practice. Monitoring quality indicators allows the identification of these gaps and, in consequence, enables specific interventions for improvement. In January 2012 began, in a Brazilian private hospital, the implementation of the Clinical Care Program (CCP) for acute myocardial infarction (AMI). The present study aims to evaluate the impact of the CCP in quality indicators for AMI including in-hospital mortality.

## Methods

All patients with a confirmed diagnosis of AMI (with or without ST elevation), after signing consent, become part of the CCP, and all care would be managed by a dedicated nurse who mobilizes a multidisciplinary team, checking records, organizing and monitoring indicators. Four indicators that are part of the Joint Commission International Library of measures were monitored before and after the implementation of the program: administration of acetylsalicylic acid (ASA) in the first 24 hours; prescription of ASA, β-blockers and ACEI/ ARB at discharge, excluding patients with contraindications. In-hospital mortality was also assessed. A comparison of the year before (Group I - 2011) versus the year after (Group II - 2012) initiation of the CCP was made. Statistical analysis included the calculation of point estimates and 95% CIs. Comparison of categorical variables was performed by chi-square and a two-tailed significant *P *value.

## Results

During 2011 and 2012, a total of 776 patients had the diagnosis of AMI in our service (Table 1).

**Table 1:** Comparison between Groups I and II

AMI patients	Group I - 2011 (*n *= 352)	Group II - 2012 (*n *= 424)	*P *value
ASA in the first 24 hours	96% (94 to 98%)	100% (99 to 100%)	<0.001
ASA at discharge	99% (97 to 100%)	100% (99 to 100%)	NS
β-blocker at discharge	94% (92 to 96%)	99% (98 to 100%)	0.001
ACEI/ARB at discharge	95% (93 to 97%)	99% (98 to 100%)	0.001
In-hospital mortality	5.1% (3.2 to 8%)	2.6% (1.4 to 4.6%)	0.06

95% CI in parentheses. ACEI, angiotensin-converting enzyme inhibitor; ARB, angiotensin receptor blockers.

## Conclusion

After beginning the CCP for AMI, a significant improvement in quality indicators occurred. Reduction of hospital mortality had a *P *value near the threshold for statistical significance.

